# Equine Facilitated Therapy for Complex Trauma (EFT-CT)

**DOI:** 10.1007/s40653-017-0187-3

**Published:** 2017-08-17

**Authors:** Tiffany M. Naste, Maggi Price, Jane Karol, Lia Martin, Kathryn Murphy, Jennifer Miguel, Joseph Spinazzola

**Affiliations:** 1 Justice Resource Institute, Needham, MA USA; 2 Boston College, Department of Counseling, Developmental, and Educational Psychology, Boston, MA USA; 3 Bear Spot Farm, Acton, MA USA; 4 Department of Psychology, Suffolk University, Boston, MA USA; 5 The Trauma Center at Justice Resource Institute, 1269 Beacon St, Brookline, MA 02446 USA

**Keywords:** Trauma, Complex trauma, Equine therapy, Animal assisted therapy, Child traumatic stress, Treatment

## Abstract

Emerging research suggests that Equine Facilitated Psychotherapy (EFP) may be beneficial for traumatized youth. In addition, complex trauma (i.e., multiple and/or prolonged developmentally adverse traumatic events which are typically interpersonal in nature) treatment research is still growing and there is a need for the development and examination of novel treatments for youth with complex trauma histories. The current article describes a promising EFP model for this population called Equine Facilitated Therapy for Complex Trauma (EFT-CT). EFT-CT embeds EFP practices within Attachment, Regulation and Competency (ARC), an extant evidence-based complex trauma treatment framework for children and adolescents. The authors provide three case studies using both observational data provided by clinicians, as well as longitudinal measures of psychosocial functioning, to illustrate the potential promise of EFT-CT. The article concludes with a discussion about implications for EFP treatment and research.

## Treatment for Complex Trauma in Youth

Initially developed to characterize specific psychiatric consequences of adult exposure to high magnitude traumatic events, the diagnosis of Posttraumatic Stress Disorder (PTSD) has been identified to be present in just over one-in-four treatment-seeking, trauma-exposed children (Huang et al. 2017). In contrast, the majority of trauma-exposed children and adolescents in a large national treatment-seeking sample have been found to manifest five prominent areas of impairment: affect regulation, attention/ concentration, impulse control, self-image, and aggression/risk-taking; with approximately one-third of these youth exhibiting prominent difficulties with attachment, somatization, sexualized behaviors and dissociation (Spinazzola et al. 2005b). The construct of *complex trauma* in children and adolescents was first articulated in 2002 by a special taskforce of the National Child Traumatic Stress Network to reflect this clinically broader and evolving array of self-regulatory, relational and attributional deficits observed in children exposed to multiple-type or recurrent maltreatment, exploitation and neglect (Cook et al. 2005; National Child Traumatic Stress Network 2017).

In contrast to proposed diagnoses offering particular symptom constellations associated with chronic interpersonal trauma exposure (most prominently for children: Developmental Trauma Disorder, van der Kolk 2005; for adults: Complex PTSD, Cloitre et al. 2011, 2012) complex trauma is not intended as a diagnosis. Rather, the complex trauma construct was intentionally designed to capture the intertwined relationship between adverse caregiving and victimization experiences and subsequent survival-based adaptations that alter normative developmental trajectories across the lifespan (Cook et al. 2005; Grossman et al. 2017; Spinazzola et al. 2013).

Clinical research on outcomes of treatment models with trauma-exposed children and adolescents has proliferated over the past decade (for a review, see Ford and Courtois 2013). Some early treatment studies for traumatized youth examined modified interventions originally designed for adults (e.g., Eye Movement Desensitization & Reprocessing (EMDR), Rodenburg et al. 2009). The majority of treatment models for trauma-exposed children and adolescents have been designed to focus upon resolution of symptoms of PTSD. Among these, Trauma-Focused Cognitive Behavioral Therapy (TF-CBT; Cohen et al. 2006) has been subjected to the most extensive empirical validation in the treatment of childhood PTSD (Cary and Mcmillen 2012; Cohen et al. 2004) and has demonstrated preliminary support in use with complexly traumatized youth (Cohen et al. 2012). Nevertheless, randomized controlled trials with child trauma populations have yet to examine either the efficacy or effectiveness of extant evidence-based models in addressing the broader array of functional and developmental impairment associated with complex trauma (Spinazzola et al. 2005a).

Initial support is emerging for a growing number evidence-based and promising practices specifically designed to address complex trauma in youth populations. On one end of the spectrum (from broadest to most narrowly focused), *Trauma Systems Therapy* (TST; Saxe et al. 2015) is a system-level intervention for complexly traumatized youth for which outcomes have been found to predicated upon of degree of involvement of layers of adults involved in the child’s caregiving system, including parents, teachers, program personnel, child welfare case managers, neighbors and community (Murphy et al. 2017). *Attachment, Regulation & Competency* (ARC; Blaustein and Kinniburgh 2010) is a well-supported, clinical objectives-driven, component-based intervention and comprehensive framework for young children through young adults and their families impacted by complex trauma (see Method section below for description and review of evidence-base). *Real Life Heroes* (RLH; Kagan 2017) is another component based approach that integrates music, expressive arts and other multi-model approaches to assist children and adolescents in creating a narrative predicated around the “hero’s journey,” a strength-based reframe of their life narrative and overcoming of trauma and adversity. RLH emphasizes ongoing identification and participation in treatment of an adult role model (in addition to the child’ primary caregiver). It has demonstrated positive outcomes in open trial pilot testing (Kagan et al. 2008).

On the other end of the spectrum of youth complex trauma interventions, *Trauma Adaptive Recovery Group Education and Therapy* (TARGET; Ford and Russo 2006) is a highly evidence-based, time-limited group or individual psychotherapy session protocol for adolesent and adult victims of complex trauma and polyvictimization (Ford 2015). TARGET is a cognitively-driven approach to emotion regulation for traumatized youth and adults that emphasizes psychoeducation about the body’s alarm system, mentalization and refocusing in effort to recalibrate trauma victims’ survival response to perceived threat. In a similar vein, *Structured Psychotherapy for Adolescents Responding to Chronic Stress* (SPARCS; DeRosa and Pelcovitz 2009) is a 16-sesion group psychotherapy protocol for adolescents that heavily emphasizes identification of maladaptive coping and cultivation of adaptive coping skills through practice of emotion regulation and mindfulness techniques. SPARCS has demonstrated initial empirical support across multiple open trials (Habib et al. 2013).

## Complementary and Integrative Treatments for Traumatic Stress

Recent research on traumatic stress and its treatment suggest that novel and nontraditional forms of therapy may be advantageous for some survivors (Metcalf et al. 2016). In particular, evidence supporting the effectiveness of mind-body and behavioral medicine techniques-- including mindfulness, sensory integration and biofeedback-based approaches-- as components of trauma intervention is growing (Dutton et al. 2013; Kaiser et al. 2010; Kearney et al. 2012; Lande et al. 2010; Tan et al. 2011). Applied neuroscientific theory coupled with anecdotal but extensive clinical observation of body-oriented treatments has highlighted the importance of somatic regulation in complex trauma recovery, particularly when utilized with treatment-resistant individuals who have been at best minimally responsive to traditional forms of trauma-focused psychotherapy (Levine 1997; Ogden et al. 2006a; van der Kolk 2014;).

Our research calls attention to three complementary and integrative forms of intervention that appear to contribute to the mitigation of some of the most challenging manifestations of complex trauma adaptation, including internalizing symptoms and behaviors, somatic dysregulation, executive dysfunction and clinical dissociation. *Trauma Center Trauma Sensitive Yoga (TCTSY)* is the first primarily yoga-based intervention to achieve inclusion in the Substance Abuse and Mental Health Service Administration’s (SAMHSA) National Registry of Evidence-based Practices and Programs (NREPP) (Emerson and Hopper 2011). TCTSY has been found to significantly reduced PTSD symptom severity and achieve high rates of loss of PTSD diagnosis in civilian females with chronic treatment-resistant PTSD (van der Kolk et al. 2014). An extended version of the TCTSY protocol has been found to also achieve significant reduction of symptoms of clinical dissociation with this population (Price et al. 2017). TCTSY has also been successfully implemented with complexly traumatized youth with severe emotional and behavioral disturbance in residential treatment settings (Spinazzola et al. 2011).

Brain biofeedback, or *clinical neurofeedback* (NFB), has been demonstrated in two recent randomized controlled trials-- one with chronically traumatized adults and a second with children with complex trauma exposure and adaptation profiles-- to be associated with significant reductions in PTSD symptoms and diagnosis, externalizing as well as internalizing symptoms and behaviors, executive dysfunction, and alexithymia (Gapen et al. 2016; Hodgdon et al. 2015; van der Kolk et al. 2016). Finally, *Sensory Motor Arousal Regulation Treatment* (SMART), the first sensory motor and sensory-integration based psychotherapy to be included by SAMHSA in NREPP, is a component-based intervention that embeds occupational therapy techniques within a phase-based trauma treatment framework for young children through adolescents, with incorporation of parent-child dyadic work (Warner et al. 2013). A match controlled study of SMART for youth with complex trauma in residential treatment settings demonstrated significant reduction of internalizing symptoms and behaviors including somatic complaints (Warner et al. 2014). Notably, somatically-oriented treatments can manifest in a variety of other forms, and may involve the use of animals to facilitate bodily regulation.

### Animal-Assisted Interventions for Trauma

Animal-assisted intervention (AAI) can be broadly defined as any therapeutic treatment that involves an animal (Kruger and Serpell 2010). AAI has been used to treat a variety of mental health difficulties, and emerging research suggests that it may be helpful in the treatment of depression (Souter and Miller 2007), anxiety (Nimer and Lundahl 2007), autism spectrum disorders (O’Haire 2013), and dementia (Filan and Llewellyn-Jones 2006). A recent systematic review of extant empirical research on its use for posttraumatic stress suggests that AAI is a promising complementary intervention for survivors of trauma, including traumatized youth (O’Haire et al. 2015).

While extant empirical examinations of AAI for posttraumatic symptomatology are limited in methodological rigor (e.g., many studies are quasi-experimental, lack randomization and/or a control group, have small sample sizes), results have highlighted the myriad mechanisms by which animals may help address symptoms associated with trauma exposure. For instance, animals are purported to reduce anxiety and hyperarousal given research indicating that their presence is related to the secretion of oxytocin (Beetz et al. 2012). Exposure to animals has been shown to be associated with increased social interaction (Wood et al. 2005) and the reduction of loneliness (Banks and Banks 2002), suggesting that AAI may reduce isolation. Research indicates that animals can elicit positive emotions (O’Haire et al. 2013), and thus may combat emotional numbing. Animals may also counteract intrusive symptomatology as they may remind survivors that danger is not present (Yount et al. 2013), and have been shown to facilitate mindful present-focused experiences (O’Haire et al. 2015; Parish-Plass 2008).

Notably, most research on AAI for trauma survivors involves dogs (e.g., service dogs), and many studies incorporated dogs into traditional clinical settings (e.g., outpatient psychotherapy; e.g., (Dietz et al. 2012; Hamama et al. 2011). However, a few studies have examined the utility of incorporating farm animals, including horses, into therapy for traumatized persons. Additionally, in a recent review of emerging interventions for posttraumatic stress disorder (PTSD), equine therapy was nominated as a popular, novel and/or emerging intervention (Metcalf et al. 2016).

### Equine-Facilitated Therapies for Trauma

Equine-facilitated psychotherapy (EFP) can be defined as the use of a horse in a therapeutic context involving a registered mental health practitioner who engages the horse to facilitate psychological and social insights (Lentini and Knox 2015). The vast majority of research on EFP examines its utility as a treatment for youth (Kendall et al. 2015), and in particular, youth identified as being “at risk” or who have been diagnosed with an autism spectrum disorder (Lentini and Knox 2015). In addition to the aforementioned benefits of AAI, EFP is purported to be an advantageous therapeutic modality through its enhancement of the therapeutic alliance between the client, therapist, and horse, and is believed to improve interpersonal adaptation skills, and foster positive attachment and resiliency (Kendall et al. 2015). EFP typically involves mounted (e.g., sitting on a horse with or without a saddle, balancing on the horse, riding with closed eyes) and/or non-mounted (e.g., observing the horse in pasture, leading a horse, grooming and bathing a horse) activities (Lentini and Knox 2015). In addition, EFP often involves more traditional psychotherapeutic techniques such as the therapist asking the client questions to facilitate introspection, encouraging positive play activities, aiding in skill acquisition, teaching communication skills, and supporting empathy development (Kirby 2010; Trotter 2012). It is also possible that EFP is advantageous for trauma survivors due to the sense of mastery one may attain through client-directed touch and skill-building, the relative absence of interpersonal triggers, as well as the co-regulation facilitated by horse-client interactions.

Five studies have been identified as examining the effectiveness of EFP for trauma survivors across multiple systematic reviews of EFP literature (Lentini and Knox 2015; O’Haire et al. 2015). One of these is a case study of one adult with Posttraumatic Stress Disorder (PTSD) (Nevins et al. 2013) and the others focus on traumatized youth (Kemp et al. 2014; McCullough et al. 2015; Signal et al. 2013; Yorke et al. 2013). In samples of child sexual abuse survivors, EFP was shown to reduce symptoms of depression (Signal et al. 2013), as well as posttraumatic stress, anxiety, and externalizing behavior problems (Kemp et al. 2014). Notably, Signal et al. (2013) emphasize that the effect sizes associated with reductions in internalizing symptoms found in their study are larger than those found in TF-CBT research. In addition, studies of youth with a PTSD diagnosis who underwent EFP evidenced reductions in PTSD symptomatology (McCullough et al. 2015) and inconclusive findings related to cortisol levels (Yorke et al. 2013). While all of these studies had small sample sizes (ranging from 4 to 30) and did not involve control groups or randomization, preliminary results suggest that there are many potential benefits associated with EFP for traumatized youth, including those with complex trauma histories (e.g., child sexual abuse). As such, EFP may be considered a promising auxiliary treatment for traumatized youth.

Despite the promising findings evidenced in research on the utility of EFP for youth trauma survivors, the literature base lacks detailed information about particular modalities, such that existing studies are not replicable and existing treatments have not been manualized (O’Haire et al. 2015). In addition, there is no uniformity across the wide variety of extant EFPs, and many do not offer a set of generalizable techniques, thus limiting treatment dissemination. Finally, existing EFP treatments for trauma are limited with respect to their integration of existing evidence-based frameworks.

The current article seeks to address this gap in the literature by providing details about EFT-CT as well as its evidence-based underpinnings. EFT-CT is a promising new intervention that incorporates elements from an evidence-based treatment framework for youth survivors of complex trauma and equine-facilitated therapies. The current article provided a concise overview of complex trauma treatment and trauma-focused equine-facilitated therapies, and will subsequently introduce the tenets of EFT-CT. The article will conclude with preliminary support for its use illustrated by three case studies, and provide an overview of the implications of EFT-CT for our growing understanding of effective treatment for complex trauma.

## Methods

### Participants

The current sample data were collected from a mental health program of the Justice Resource Institute (JRI). JRI provides residential, in-home, and community-based services to youth and adults with a multitude of challenges (e.g., social, emotional, behavioral difficulties) across diverse populations in Massachusetts, Rhode Island and Connecticut. The Community Based Service (CBS) Division of the agency primarily serves families and children. Many of the families serviced by the Boston CBS programs are living in an urban setting, and live at or below the poverty line. These families often face ongoing community violence, lack resources, and endure structural inequalities that impact development and functioning.

### History of Model Development

JRI seeks to provide intensive, flexible, culturally responsive, individualized, community based, and family-focused services. In addition, JRI utilizes a trauma-informed framework called Attachment, Regulation and Competency (ARC). ARC is an evidence-based treatment framework for youth who have experienced complex trauma (Blaustein and Kinniburgh 2010). ARC addresses multiple domains of impairment associated with complex trauma symptomatology. Importantly, ARC has been shown to be effective in the reduction of PTSD symptoms, internalizing difficulties, and externalizing behaviors in youth in diverse treatment settings (e.g., residential, outpatient; Arvidson et al. 2011; Hodgdon et al. 2016; Hodgdon et al. 2013; IFC MARCO 2010). In response to the multifaceted presentations of the youth and families served, JRI sought to explore alternative treatment models that target the domains and subskills of ARC. These include alternative forms of therapeutic interventions such as yoga, meditation, and animal assisted therapies.

EFP was chosen as one alternative intervention that could be further developed and refined to be trauma-informed and complimentary to extant clinical work at the agency. This exploration resulted in a partnership with The Bear Spot Foundation for Equine Facilitated Psychotherapy, which provides therapeutic services to children, adolescents, and their families. Together, JRI and the Bear Spot Foundation integrated existing EFP practices with the ARC model, resulting in EFT-CT. Subsequently, clinicians at JRI’s Boston CBS program identified youth to participate in the pilot to test this model.

### EFT-CT Model Description

EFT-CT is a trauma informed ARC-based intervention that can be used alone or in conjunction with more traditional forms of psychotherapy. Consistent with the ARC framework, EFT-CT incorporates 3 core components of intervention that target areas impacted by exposure to trauma including: 1) *safety*, 2) *attachment*, and 3) *regulation*. In addition, *routines and rituals* (i.e., treatment techniques that create an environment reflective of safety, predictability, and consistency) are woven throughout the model.

*Safety* is the first core concept, and serves as the foundation for all EFT-CT work. Safety refers to a shared sense of relational safety between the client and horse, which is theorized to foster treatment engagement, positive attachment, and relationship building. Safety is a critical component in complex trauma treatment, and some argue that treatment for this population may even be harmful if safety is not addressed at the outset (Courtois 2004; Pearlman and Courtois 2005). More specifically, safety is enhanced through a comprehensive introduction to the farm/facility, and safe and correct interactions with the horse and related equipment. To incorporate routines and rituals, sessions begin with greeting the horse, leading the horse into the barn, and grooming the horse.

It is likely that part of the therapeutic success of EFT-CT is due to the incorporation of safety regulations imperative to working with horses. Such work can teach clients how to stay safe with horses, which may subsequently generalize to their outside lives. For example, the client learns how to act around the horse in a way that demands clear boundaries and respect from the horse, and in turn learns how to treat humans in similar ways. Once a foundation of safety and mutual respect (between horse and client) is achieved, there is ample opportunity for the client to experience safe touch and physical contact with the horse. In this way, a client who has suffered severe trauma can learn, using the same elements in his or her relationship with the horse, how to create safe and trusting relationships with humans involving safe and nurturing physical contact.

The second core concept, *attachment,* focuses on relationship building. This concept is essential, as traumatized youth often have difficulties forming and sustaining healthy and meaningful relationships. The two primary components within attachment are 1) *caregiver affect management,* and 2) *attunement*. Clinicians utilize effective caregiver affect management strategies (e.g., coaching, modeling appropriate interactions with the horse) to support the client, which in turn allow the client to experience and respond to the emotions of the horse in a non-threatening context. Through this process, the client learns how to understand, or attune to, the non-verbal cues given by the horse, as well as how to react to these cues effectively. The immediate and uncomplicated response of the horse to the client’s behavior serves as a quick and effective teaching tool. This process helps the client develop effective and empowering non-verbal communication skills with the horse. As the client gains mastery in attunement and communication with the horse, his or her attachment to the horse deepens. Once effective communication is established, the client can learn about and attend to the emotional world of the horse. In turn, the horse begins to focus on the client, respond to the client’s interactions, and consequently develop trust. These new communication skills, and resulting attachment, can then be generalized to human relationships.

The third core concept is *regulation*, which includes 1) *body awareness*, 2) *co-regulation* and *3) rhythm*. Bodily dysregulation is a common feature of complex trauma symptomatology and thus techniques to improve body awareness are often incorporated in treatments for this population (e.g., Ogden et al. 2006b; Warner et al. 2013). In addition, the use of one’s body is the sole tool for communication between humans and horses, and thus *body awareness* is critical in EFT-CT. As the client learns how his or her gestures and postures impact the horse’s responses, the client can then generalize these lessons to communication with friends and family. By mirroring the clinician, the client can learn how to regulate physical reactions to improve effective communication with the horse. For example, a horse may at first fear a client’s quick impulsive movements. The client will then learn to slow his or her body to result in more relaxed responses from the horse. These thoughtful, non-impulsive movements also require the client to engage executive functioning skills.

The client is then able to *co-regulate* by fully accessing the horse’s natural rhythms through effective bodily communication. Co-regulation is a critical component of treatment for complex trauma and is emphasized in multiple evidence-based models for traumatized youth (e.g., Arvidson et al. 2011; Becker-Weidman and Hughes 2010), as well as novel treatment models for complexly traumatized children (Blaustein and Kinniburgh 2010). As the client is guided to use his or her body differently, and experiences the horse’s emotional state change in reaction, the client’s internal affective state will also transform. Ultimately, these repetitive positive experiences with the horse and clinician support long-term effective body modulation strategies. Finally, the natural *rhythm* of the horse’s three gaits (walk, trot and canter) can help the client access his or her own internal rhythms. As the client rides, the horse’s natural rhythms can help the client access their own meditative re-centering rhythms both on and off the horse. In addition, the clinician can help the client associate these rhythm states with a word that they can later recall, helping them access these rhythms when experiencing negative emotional escalation in other environments.

### Present Study Design

The current study employs empirically-driven clinical case outcome methodology using longitudinal data derived from an agency-designed clinical quality improvement database (see below for more details). More specifically, three clinical cases of youth who completed EFT-CT will be presented. These cases were derived from a larger EFT-CT pilot (N ~ 20) that is currently in progress. Case descriptions will include both narrative information provided by clinicians, as well as objective data derived from standardized measures of psychosocial functioning across a variety of domains associated with complex trauma symptomatology. All Institutional Review Board procedures were followed in this study, and illustrative cases were de-identified to ensure protection of human subjects.

The current study analyzed data from the Client Assessment Tracking System (CATS). CATS is a web-based interface that is used to gather clinical outcomes data in a regular and systematic way for the purpose of continuous quality improvement. The CATS system is modeled after the NCTSN CORE Data Set and has been implemented at all JRI programs, resulting in longitudinal data collection for over 2400 youth and families thus far. All JRI administrators, clinical staff, and a subset of milieu staff are trained in the administration and reporting of clinical outcomes. The CATS system includes a comprehensive battery of reliable, well validated, trauma-informed psychometric instruments that can be utilized with children ranging from 1.5 years to 21 years of age.

### Measures

CATS includes a battery of standardized self-report and clinician- or caregiver-rated measures designed to provide a comprehensive evaluation of the six core domains that may become impaired by exposure to chronic, interpersonal, or complex trauma. These include biological regulation (e.g., somatization, sensory motor difficulties, alteration of pain thresholds), affect regulation (e.g., alexithymia), dissociation, behavioral control (e.g., poor modulation of impulses, self-harm, difficulty following rules), cognition (e.g., attention regulation, executive functioning), self-concept (e.g., self-esteem), and attachment (e.g., interpersonal difficulties, problems within care-giving relationships). In addition to assessing information about client demographics, the database includes the following clinical assessment measures:

#### UCLA-PTSD Stress Reaction Index

(PTSD-RI; Pynoos et al. 1998) is a client self-report measure of DSM 5 posttraumatic stress symptoms experienced in the past month. The measure includes 31 items that assess the presence and frequency of post-traumatic stress symptoms, rated on a Likert scale ranging from 0 (none of the time) to 4 (most of the time). Twenty-seven of the items map directly onto PTSD diagnostic criteria, and can be broken down by the 4 DSM symptom clusters (intrusions, avoidance, alterations in cognitions and mood, alterations in arousal and reactivity); four additional items assess for the presence of dissociation. Items are scored to produce a continuous score indicating overall trauma symptomatology, with higher scores indicating more symptoms.

#### Abbreviated Dysregulation Index

(ADI; Mezzich 2001) is a 30-item self-report instrument measuring dysregulation. Items are rated on a four-point Likert scale with the following range: Never, Occasionally, Mostly, and Always. There are three subscales within the ADI: cognitive dysregulation, affective dysregulation, and behavioral dysregulation. Each subscale consists of ten items, and is deemed clinically significant if at least five out of the ten items are rated “Mostly” or “Always”. The measure has strong internal consistency, with a Cronbach’s alpha ranging from 0.71 to 0.93 for the three subscales (Mezzich 2001).

#### Children’s Depression Inventory

(CDI-2; Kovacs 2010) is a 28-item self-report instrument measuring the extent and severity of depressive symptoms for children ages 7–17 years. For each question, the respondent selects which of three statements best represents how he/she has felt in the last 2 weeks; each statement corresponds to a rating ranging from 0 to 2. Responses are summed to produce a total depression score indicating the severity of depressive symptomatology. The CDI-2 is interpreted using t-scores based on age and sex-based norms, and include a cut-off score (*T* = 65) to determine if depressive symptoms are clinically significant. The CDI-2 is a nationally normed measure that demonstrates adequate to high internal consistency across the total scale and subscales for all ages, with Cronbach’s alphas ranging from 0.67 to 0.91 (Bae 2012)

#### Child Dissociation Checklist

(CDC-3; Putnam 1997) is a 20-item parent-report measure of children’s dissociative symptoms. Respondents rate the accuracy of statements that describe the child’s behavior over the past 12 months using a three-point Likert scale, where 0 represents “not true”, 1, “somewhat or sometimes true”, and 2, “very true”. The total score is calculated as the sum of all individual item scores, with higher scores reflecting more severe dissociative symptomatology. The CDC has strong internal consistency (Cronbach’s alpha of 0.95; Putnam 1997).

#### Adolescent Dissociative Experiences Scale

(A-DES; Armstrong et al. 1997) is a 30-item self-report measure that assesses multiple dimensions of dissociation in adolescents ages 12–18 years. Items are rated on an 11-point Likert scale ranging from 0 (“never”) to 10 (“always”). A-DES total scores are computed by summing all endorsed items, and dividing by the total number of items endorsed, with higher scores indicating more severe dissociative symptoms. Initial assessment of psychometrics of the A-DES indicated strong internal consistency, with a Cronbach’s alpha of 0.93 (Armstrong et al. 1997).

#### Somatic Awareness Measure

(SAM; Stone 2014) is an internally developed self-report instrument used to assess dimensions of bodily and somatic awareness. The measure includes 24 items rated on a five-point Likert scale ranging from 0 (“Never”) to 4 (“All the time”) and yields scores ranging from 0 to 32 on three subscales: Somatic Sensory Sensitivity, Somatic Problems, and Body Awareness, each of which is interpreted slightly differently. Somatic Sensory Sensitivity assesses awareness of sensory input from physical sensations such as pain, heat and hunger; very high scores and very low scores are both problematic, indicating difficulties with hypersensitivity and hyposensitivity, respectively. Somatic Problems assesses problems with bodily functions and bodily pain; high scores on this subscale indicate more severe somatic difficulties. Body Awareness assesses one’s awareness of bodily manifestations of emotional states; higher scores on this subscale indicate better functioning (e.g., more awareness of physical signs of emotion). An initial assessment of the psychometric properties of the SAM indicated strong internal consistency, with a Cronbach’s alpha of 0.875 (Stone 2014).

#### Child Behavioral Checklist

(CBCL; Achenbach and Rescorla 2001) is a 120-item caregiver report of functioning and behavior problems for youth ages 6–18 years. Items are responded to using a 3-point Likert scale system (0 = not true; 1 = somewhat/sometimes true; 2 = Very true/often true). The measure yields eight empirically derived syndrome scales and six DSM-oriented scales that align closely with common DSM diagnostic categories. The CBCL includes age and sex-based norms, with T-scores of 65–69 representing the subclinical range and T-scores of 70 and above signifying the clinically significant range for the individual subscales. The subscales can be reduced to two broadband scales of internalizing and externalizing problems and summed to produce a total problems score (T-scores of 60–64 indicating subclinical range; T-scores of 65 and higher indicating clinical range). The CBCL is one of the most frequently used assessment measures with a large body of research supporting its psychometric validity.

#### Behavior Rating Inventory of Executive Functioning – Parent

(BRIEF-P; Gioia et al. 2000) is an 86-item observer-report of youth exectutive functioning. Each item is responded to using a 3-point Likert scale (never, sometimes, often). The measure yields two theoretically and statistically derived index scales of executive functioning: 1) Behavioral Regulation, which breaks down further into subscales measuring inhibition, shifting and emotional control; and 2) Metacognition, which can be further reduced to initiaion, working memory, planning/organizing, organization of materials and self-monitoring. The items can also be summed to yield a Global Composite of executive functioning. T-scores of of 65 or higher are considered clinically significant on this measure. The BRIEF is a well-normed and validated measure, with Cronbach’s alpha coefficients ranging from 0.80 to 0.98 (Gioia et al. 2000).

#### Children’s Alexithymia Measure

(CAM; Way et al. 2010) is a 14-item parent-report measure that assesses the degree to which a child or adolescent ages 5–17 years is having difficulty recognizing and expressing feelings. The CAM uses a 4-point Likert rating scale; items are summed to yield a single total score of alexithymia ranging from 0 to 42, with higher scores indicating more difficulty with alexithymia. Preliminary examination of the psychometric properties of the CAM demonstrated strong internal consistency, yielding a Cronbach’s alpha of 0.92 (Way et al. 2010).

All of the above measures are completed every three months, and can only be entered during pre-programmed windows of time to ensure that changes in clinical presentation are accurately assessed. Each measure is scored immediately using the validated scoring systems designed by the author/publisher of each scale, yielding raw scores, T-scores, and/or percentile scores.

## Results

### Preliminary Support for EFT-CT: Case Studies

#### Case 1: Mia

Mia is a 10-year-old African American female living with a family member in an urban area. Mia is diagnosed with PTSD due to abuse and neglect experienced while living with a foster family. Mia was born addicted to substances and consequently taken into custody by the Department of Children and Families (DCF) and placed with a foster family who fled to another state. During this period, Mia was subject to extensive abuse and neglect by her foster parents, including severe sexual abuse. Mia was subsequently brought back to the area in which she was born, and placed with a family member with whom she currently lives. Mia presents with sexualized behaviors which she has acted out towards family members and the family dog. She also has a history of isolated incidents during which she plays with her feces.

Mia is an intelligent and bubbly girl that can present with a high rate of distractibility and impulsivity. Mia demonstrates oppositional behaviors when teachers or providers attempt to redirect her, but she is often able to reflect on/has insight around her actions. Mia has been receptive to trauma-informed interventions, which is why she was initially referred for equine therapy. Mia is motivated by working with animals and gravitates towards “hands on” activities.

Before the start of EFT-CT, Mia was highly symptomatic across a range of clinical domains. On the CBCL, her primary caregiver indicated that Mia exhibited a number of behavior problems on the (CBCL Total Problems T-score = 68, clinical range) both internalizing and externalizing in nature, including clinically significant social problems (*T* = 72), thought problems (*T* = 74), and attention problems (*T* = 77). Her caregiver also reported broadly impaired executive functioning on the BRIEF-P, indicated by a Global Executive Composite score in the clinically significant range (*T* = 71, 98th percentile), and with the most significant problems in the domain of metacognition (*T* = 75, 98th percentile, clinically significant range). In addition to significant difficulty with getting started on tasks, organizing her thinking, or strategizing, self-monitoring and working memory, Mia exhibited significant difficulties with behavioral inhibition (*T* = 78, 98th percentile). Regarding trauma-specific symptomatology, Mia endorsed clinically significant problems in two of the three symptom DSM-IV-TR symptoms clusters on the PTSD-RI (avoidance, *T* = 80; arousal, *T* = 92), as well as subclinical problems with trauma-related intrusions (*T* = 63), and a posttraumatic stress total score also in the clinically significant range (*T* = 86). She also endorsed clinically significant problems with dissociation (*T* = 75), and sexual concerns (*T* = 99), related to Mia’s trauma exposure. Mia herself did not endorse any significant symptoms on a self-report of depression (CDI-2 total problems T-score = 54; 66th percentile).

Mia was able to form a strong and positive attachment with the therapy pony during EFT-CT sessions. Because of her motivation to work closely with the pony, she successfully followed instruction and safety protocols. Much of the work with Mia at Bear Spot Farm focused on riding and helping her gain an understanding and awareness of her body. For instance, her clinician carried out rhythm and balance-based exercises with Mia while she rode, to help her gain an effective riding position, and thus communicate with therapy pony better. These exercises set the stage for Mia to be in her optimal window of arousal, evidenced by her decreased impulsivity and heightened sense of control over her body. Mia demonstrated improved functioning and was able to express feeling “free” and “calm” while she rode.

Due to difficulties with transportation and scheduling, Mia was unable to continue her participation in the pilot program. While she continued to receive other services, Mia demonstrated setbacks after exiting the pilot program. Increased impulsivity and oppositional and unsafe behaviors have created challenges for Mia and her family across multiple domains. After treatment, Mia also played with and hid her feces on a few occasions. Mia did not complete enough treatment to reach a second clinical assessment period, thus, no data on her clinical functioning post-treatment is available.

#### Case 2: Kari

Kari is an 11-year-old Caucasian female who resides in an urban area with her adoptive parents and sibling. Kari has been diagnosed with PTSD, Reactive Attachment Disorder, and intellectual impairment. Prior to adoption, Kari lived with her biological mother and father during which time she was subjected to multiple forms of abuse and neglect. Kari’s biological mother used substances while pregnant, both of Kari’s biological parents were frequent substance users, and her father was incarcerated when Kari was three years old. Prior to adoption, Kari was briefly involved in the foster care system after living in a shelter with her biological mother. Kari’s ongoing trauma symptoms are indicative of severe sexual abuse.

Kari is a kind and nurturing girl who presents with a high rate of distractibility and hyperarousal. Kari becomes dysregulated easily, evidenced by sudden increases in energy, difficulties with boundaries and bodily awareness, and oppositional behavior. Kari often seems unaware of bodily sensations, which inhibits her ability to self-regulate. Though she has an established support network at school, she struggles to initiate and maintain healthy peer relationships. Kari exhibits significant difficulties with interpersonal engagement and communication across multiple life domains. She often becomes oppositional towards family, peers, and providers, and these behaviors have increased with age. Kari has a history of physically assaultive and sexualized behaviors directed specifically towards one of her adoptive parents; however, family reports no reoccurrence of these behaviors in the three years prior to treatment onset.

Prior to the start of treatment, Kari’s parents reported problems at the borderline clinical or subclinical level in several domains of the CBCL, including social problems (*T* = 68); anxiety Problems (*T* = 68); oppositional defiant problems (*T* = 63); anxious/depressed Problems (*T* = 62); and affective Problems (*T* = 60). These subscale elevations fell mostly in the internalizing domain (CBCL Internalizing scale *T* = 60, subclinical range), and were consistent with her clinical presentation of chronic low self-esteem, social difficulties and anxiety. With respect to executive functioning, her parents endorsed on the BRIEF-P clinically significant disruptions in inhibition (*T* = 70; 97th percentile), as well as subclinical difficulties with monitoring (*T* = 64; 92nd percentile), or the ability to keep track of one owns behavior and progress toward task completion.

Although Kari did not meet full DSM criteria for PTSD at baseline, on the PTSD-RI, her parents endorsed moderate PTSD symptom levels (total symptom score = 20), with the most symptoms in Cluster D (alterations in cognitions and mood) and Cluster E (alterations in arousal and reactivity). Kari’s parents denied the presence of dissociative symptoms on the CDC-3, and rated Kari as possessing normative ability to reflect upon and discuss her own emotions on the CAM. However, despite her pronounced clinical difficulties in these areas, Kari denied problems with either somatic sensory processes or affective/behavior regulation on two self-report measures (SAM and ADI). This discrepancy between parental appraisal of Kari’s capacity for reflection and Kari’s denial of known emotional and sensory difficulties raised questions about the actual extent of Kari’s emotional awareness or self-insight.

Kari made significant gains while participating in EFT-CT for two years. For approximately the first 18 months, sessions involved Kari retrieving the therapy horse from his paddock, leading him into the barn, and carrying out a grooming routine. Effective grooming requires rhythmic and consistent physical engagement using a series of brushes. While grooming, Kari became regulated and able to engage conversations with her therapist at a level of clarity, organization and fluency beyond that which she was typical capable in her traditional in-home or clinic-based psychotherapy sessions.

Over time, Kari’s tolerance for more difficult conversations about her emotions, social difficulties and energy level also increased during these sessions. This ground work also resulted in a strong attachment between Kari and the therapy horse. In-home and outpatient providers worked with Kari to generalize the relational skills Kari has acquired through this bond to her social engagement and interactions with peers, teachers and family.

Eventually, Kari expressed feeling comfortable enough to begin riding. It took roughly one and a half years of consistent ground work with a therapy horse before she felt safe enough to ride. Through her riding, Kari is able to experience a variety of rhythms as the horse transitions between gaits (walk/trot). This has allowed her to gain an understanding of her body as it pertains to effective riding position and further communication with the therapy horse. Kari has also been able to practice energy modulation in the moment, by asking the horse to move faster or slower. These cues are effectively transmitted to the therapy horse through different types of physical contact initiated by the rider. Kari has had great success with riding, and continues to gain a sense of empowerment from EFT-CT sessions.

The clinician symptom scores collected throughout the duration of Kari’s course of treatment reflect the clinically observed gains described above, but also indicate a non-linear pattern of improvement across a number of domains. The first notable change that occurred was in regard to Kari’s own capacity to reflect on her affective and behavioral functioning. For example, although Kari initially denied having difficulty with behavioral, emotional, or cognitive regulation on the ADI, after three months, she endorsed significant difficulties with both affective regulation (i.e., the ability to modulate the expression and intensity of her emotions) and behavioral regulation (i.e., the ability to control one’s own behavior). Similarly, she initially denied having difficulties with body awareness on the SAM, but by the second assessment period, Kari endorsed significant problems recognizing how her emotions manifest in her body (SAM Body Awareness raw score change from 12 to 3). Recalling that higher scores on the Body Awareness scale of the SAM are indicative of higher functioning and better overall awareness of how one’s emotions manifest in their body, these shifts could be interpreted as reflecting an actual increase in distress or decrease in psychological functioning, perhaps in response to a situational stressor. However, given the consistency of these self-reported ratings with caregiver and clinician reports at baseline, it is more likely these changes reflect increased insight into self, or a new willingness in Kari to acknowledge her difficulties with bodily awareness. As the affective and behavioral dysregulation problems began to decrease, and Kari became more regulated during treatment, she was better able to notice her difficulties in the area of cognitive regulation, or the ability to plan, organize, anticipate outcomes, and learn from experience (ADI cognitive dysregulation symptom count change from 2 to 6).

**Fig. 1:**
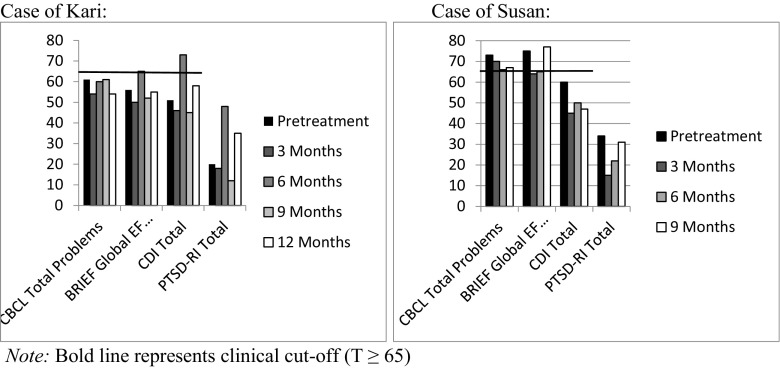
Patterns of clinical change for presented cases

In addition, there was a subtle linear change in alexithymia and behavioral problems. Throughout the duration of the intervention, Kari demonstrated consistent improvement in her ability to identify and discuss her emotions (CAM scores changed from 13 to 6 from pre-to post-treatment). Regarding her CBCL scores, almost all of the domains in which Kari had subclinical problems at baseline showed improvement throughout the course of treatment, as well. Despite these improvements, there was a notable elevation in Kari’s self-reported depression scores across all domains (CDI-2 Total symptoms *T* = 73; 99th percentile) after six months of EFT-CT treatment. However, these scores all dropped below the clinical range by the final assessment period. During this same period, there was a similar spike in self-reported PTSD symptoms (PTSD-RI total symptom score change from 20 to 48) and executive functioning difficulties, with new problems emerging in attentional shifting (*T* = 63; 92nd percentile; subclinical range) and Working Memory (*T* = 61; 88th percentile; subclinical range). Notably, these too, returned to baseline levels by the last assessment period. The pattern of change in Kari’s clinical symptoms throughout the course of treatment is illustrated in Fig. 1.

#### Case 3: Susan

Susan is a 12-year-old Latina female living with her adoptive mother and father in an urban community. Susan is diagnosed with PTSD and several learning disabilities. Susan’s biological mother is a non-biological relative of her adoptive mother. Susan was placed with her adoptive mother after being removed from her biological mother’s care at age 5 due to her biological mother’s substance abuse and prostitution in the home. On occasion, Susan was made to dance for her mother’s male clients. Susan’s adoptive family appears to be supportive, however there is ongoing tension in the home because of her behaviors.

Susan is an expressive, sociable girl that is motivated and willing to try new things. She was referred for equine therapy services to help her develop coping skills for impulsivity, and to solidify positive relationships. Susan has made incredible strides in safely learning the proper ways to handle, manage, ride, and care for the therapy horse. Susan struggles both academically and socially in school. She has made mean and derogatory statements towards peers and has become physically assaultive towards peers and staff on the school bus. In addition, she sometimes demonstrates extreme attention seeking behaviors, which ultimately result in her feeling isolated. Finally, Susan is especially tall for her age, which has resulted in bullying as well as being perceived as intimidating by her peers.

Susan’s baseline clinical measures prior to the start of treatment indicated a wide range of behavior problems as reported on the CBCL (CBC Total Problems T-score = 73), with most problems falling along the externalizing spectrum (*T* = 74). However, subclinical internalizing problems (*T* = 62) were present on the CBCL as well. Per parent report, Susan had clinically significant scores on measures of CBCL aggressive behavior (*T* = 80), social problems (*T* = 77), attention problems (*T* = 73), and thought problems (*T* = 71); and borderline clinical scores on rule-breaking behavior (*T* = 69); and anxious/depressed problems (*T* = 67). Her mother also reported a number of clinically significant problems related to executive functioning on the BRIEF-P prior to treatment. These included problems of equal concern across both the metacognition and behavioral regulation domains, resulting in a Global Executive Functioning composite score in the clinically significant range (*T* = 75; 98th percentile). Specifically, Susan exhibited the most difficulty with inhibition (*T* = 90; 99th percentile) and monitoring (*T* = 88; 99th percentile). Notably, her mother also endorsed clinically significant symptoms of dissociation on the CDC-3, with her scores consistent with a dissociative disorder. However, Susan evidenced relative strength in terms of her ability to identify, reflect on, and discuss her emotions, as indicated by the CAM.

Despite her observed difficulties with aggression, prior to treatment Susan did not endorse problems in this domain. For example, while she endorsed problems with cognitive regulation on the ADI (i.e., problems with planning, organizing, and learning from her experiences), she denied difficulties with emotion and behavior regulation on this same measure. Susan also reported some difficulties with body awareness and sensitivity on the SAM. In addition, Susan denied clinically significant depressive symptoms, but did acknowledge difficulties in two domains of the CDI-2, including ineffectiveness (*T* = 67; 96th percentile) and functional Problems (*T* = 66; 95th percentile). This indicates that while Susan was not experiencing depression as a clinical syndrome at the start of treatment, she was experiencing significantly negative views of her own abilities and academic performance, as well as difficulty effectively engaging in daily tasks. Lastly, Susan endorsed some trauma-related difficulties on the PTSD-RI, including elevated problems with altered cognitions and mood and some problems with altered arousal and reactivity. She indicated that some of her predominant concerns were that the world feels dangerous, she feels that some part of her trauma was her fault, feelings of shame, difficulty sleeping, feeling jumpy and frightened, and startling easily. However, Susan did not meet criteria for a diagnosis of PTSD at the start of her treatment.

EFT-CT sessions at Bear Spot Farm with Susan have strongly emphasized safety. Through her grooming routine and riding, she has gained insight on how her behavior impacts the therapy horse and vice versa. Susan is very motivated to ride but becomes anxious and doubtful upon mounting the therapy horse. With coaching and support, she has been able to recognize that leading the therapy horse around the arena a few times prior to getting on is helpful in reducing anxiety. Building routines and rituals specific to Susan’s needs has further allowed her to excel, providing her with a sense of competency and empowerment. With these supports, she has been able to express her feelings openly and process emotions related to her trauma history.

This was especially pertinent after a trip she took with her adoptive parents to visit her biological parents. While on the trip, Susan spent time with her biological mother and father, who were dismissive of her. Upon return to EFT-CT sessions, she disclosed this during the riding portion of her session. Once mounted on the therapy horse and comfortably walking around the arena with close support from clinician, Susan stated that she was able to see her biological parents while away, then added that it wasn’t a good time. She was able to engage in a discussion linking her unsafe and anxious feelings with her interactions with her biological parents. In this moment on the horse, it became evident that Susan is more regulated and seemingly better able to discuss difficult experiences. Susan noted that she is aware that her biological parents were never able to do a good job of keeping her safe, and that feeling unsafe exacerbates her anxiety. Susan went on to express a newfound sense of connection and appreciation for her adoptive mother who she notes is better able to keep her safe. Susan continues to show receptivity to exploring the linkage between her history and how it is affecting her today.

Clinical measures completed throughout the course of treatment indicate that Susan demonstrated some notable patterns of improvement. For example, Susan’s behavior problems steadily decreased throughout the period of intervention, as reported by her mother on the CBCL. In several domains of behavior on the CBCL, her scores dropped by over one standard deviation and at times dropped out of the clinical range, indicating significant clinical improvement. These include anxious/depressed problems (*T* = 51); aggressive behavior (*T* = 65) and anxiety problems (*T* = 55). She also made gradual but notable improvement in social problems (*T* = 68), thought problems (*T* = 64) and conduct problems (*T* = 65) on the CBCL, all of which moved from the clinically significant range to the subclinical range. In addition, her scores on scales measuring CBCL somatic problems (*T* = 59) and oppositional defiant problems (*T* = 59) moved from the subclinical range to the nonclinical range. Attention problems remained a current and significant concern, with current scores similar to those observed at baseline (*T* = 73).

Similar gradual but consistent improvement was also observed throughout the course of treatment with regard to Susan’s executive functioning. Two scores on the BRIEF-P moved below the clinical range (Shift, *T* = 57; 79th percentile, and Emotional Control, *T* = 56; 75th percentile) and many of her scores on this measure dropped one standard deviation or more over the treatment period, including inhibition (*T* = 75; 98th percentile), working memory (*T* = 64; 90th percentile), and monitoring (*T* = 64; 92nd percentile). As such, Susan’s overall Global Executive Functioning Composite improved by one standard deviation, though it remained in the subclinical range, suggesting that while she made significant and broad gains in her executive functioning abilities there is a need for continued work in this domain.

The trend of gradual and consistent improvement was also observed in symptoms of specific clinical syndromes, with self-reported problems of ineffectiveness and functional problems related to depression both dropping out of the clinical range on the CDI-2 (T-scores of 53 and 52 respectively; 61st and 58th percentiles respectively). In addition, there were self-reported decreases in total PTSD symptoms on the PTSD-RI (PTSD total symptom score change from 34 to 22) and dissociative symptoms on CDC-3 (raw score change from 24 to 7). Areas in which little change was observed throughout the course of treatment included attention problems (as indicated above), as well self-reported problems with cognitive regulation on the ADI scale, and somatic sensory sensitivity on the SAM. The pattern of change in Susan’s clinical symptoms throughout the course of treatment is illustrated in Fig. 1.

## Discussion

The present exploratory study utilized an empirically-driven, clinical case outcome methodology to introduce EFT-CT, an innovative ARC-based approach equine facilitated complex trauma intervention with children and adolescents. Approximately 20 clients have participated in the EFT-CT pilot for varied lengths of time. Participants appear to have made gains in several areas. Most notably, a decrease in symptoms associated with trauma has been evidenced. Preliminary clinical quality improvement and case-level outcome data support a decrease in anxiety, depression, somatic/sensory complaints, and behavioral dysregulation. Additionally, EFT-CT pilot participants have shown improved interpersonal skills, communication strategies, and overall social functioning. As a result of clinician support and the development of a shared sense of safety fostered by routines and rituals, prototypical participants demonstrated improved internal regulation and organization. Finally, after treatment completion, many participants evidenced higher-order cognitive functioning (e.g. accessing language, decision making) and the development of positive coping skills.

EFT-CT may play an integral role in decreasing symptoms related to trauma in children and adolescents. The case outlines above suggest that participants benefited from EFT-CT in multiple ways (e.g., reduced internalizing problems including reduction of somatic symptoms; improved affective and sensory awareness, executive functioning and interpersonal skills; reduced externalizing difficulties). Participants also demonstrated increased capacity to recognize and respect boundaries within the context of effective trust and relationship building. As clients continued to explore their relationship with their therapy horse, they appeared to become became more attuned to its needs and aware of the horse’s responses relative to themselves. Experiencing safe and positive touch also appeared to foster therapeutic progress. It seems that EFT-CT promotes feelings of safety and empowerment, which may result in part from the client’s newfound sense of mastery and competency in horse care and riding. Finally, the natural rhythms of the horse’s gaits support the client’s internal regulation which may be related to decreases in somatic dysregulation.

While observations from this exploratory study should be considered very preliminary, they are nevertheless encouraging, and suggestive of the potential for EFT-CT to function as a meaningful addition to the growing arsenal of complex trauma-focused interventions for children and youth. EFT-CT may provide an opportunity to deepen engagement of these youth in treatment in the absence of full-system involvement as is desired for optimal implementation of TST. EFT-CT may assist youth in building clinical competencies in areas of relationship building, self-reflection and self-expression that advance their readiness and capacity to engage in narrative and identity work such as that undertaken in interventions like Real Life Heroes. EFT-CT provides a somatically driven approach to regulation for children and youth who might not be ready or have the aptitude for the heavier cognitive demand of TARGET. In its focus on attachment, empathy building and attunement through dyadic engagement of a horse versus a person, EFT-CT may provide a better tolerated initial foray into relationally-based intervention for interpersonally avoidant or reactive youth than peer-driven interventions like SPARCS. EFT-CT offers a novel and compelling avenue of integrative, mind-body focused complex trauma intervention in the company of other emerging evidence-based interventions like TCTSY, NFB and SMART. This is desirable, as it increases client choice and expands options for tailoring treatment to resonate with the interests and aptitudes of specific complexly traumatized youth.

### Limitations

While participants in the current EFT-CT pilot appear to have benefited from treatment, there are a few noteworthy limitations to the current treatment. First, the current program is located in a rural area, which makes it difficult to access for participants in urban communities. This is especially true of participants with limited financial resources and/or little access to transportation. For instance, Mia had to discontinue participation because she could not obtain adequate transportation to Bear Spot Farm. Second, the cost of general care of the horses is high and third party billing is limited for such services.

With respect to EFP more broadly, there is little consensus regarding theory and technique. Lack of consistency across models limits treatment dissemination and examination. As such, the development of manualized or routinized treatment programs and/or certification programs for EFT-CT and other EFPs is critical. In addition, it is important that EFP providers are adequately trained, as horses are large, powerful, and unpredictable animals. EFP clinicians must be experienced equine professionals to ensure that treatment is safe. There currently exists relatively few EFP training programs, and thus additional resources for training are pertinent for the dissemination of safe EFP.

### Future Directions

The current study outlines promising results for EFT-CT pilot participants with histories of complex trauma exposure and adaptation. It is possible that the benefits of EFT-CT are robust enough to allow clients to subsequently begin verbally processing their traumatic experiences verbally. For instance, the client may benefit from discussing traumatic memories with the clinician while riding the horse, thereby benefiting from the simultaneous rhythm and regulation provided in this movement. The rhythm of the horse’s walk as the client finds language to express these memories would likely serve as repetitive somatic regulating system as they enter this difficult and often decentering conversation. In addition, this position allows the client to sit above the clinician which may allow the client to feel empowered.

It is necessary for these theories to be further tested empirically. Future studies will need to recruit larger samples and utilize treatment efficacy research methodologies including randomized controlled trial study design. In addition, research on the potential neurological impact of EFT-CT may enhance our understanding of the mechanisms by which EFT-CT results in positive outcomes. Such research is currently beginning underway at Bear Spot Farm, and includes an examination of meditation on compassion.

In sum, EFT-CT may be a powerful experiential therapy for traumatized youth. Current case studies suggest that EFT-CT participants experience positive changes across a variety of psychosocial outcomes. Given the novelty of research on EFPs more broadly, it is critical that further theory and research on the benefits of psychotherapy involving horses be conducted. In particular, research on use of EFPs with traumatized clients is of particular importance given the vital need of these clients for safety, positive attachment, somatic awareness and regulation, and social skill development, and the unique potential for horses to aid in the amelioration of these specific difficulties.
